# Cocoa cultivation as an engine of rural development: Impact on the configuration of livelihoods strategies of rural households in Colombia

**DOI:** 10.1371/journal.pone.0355387

**Published:** 2026-08-20

**Authors:** Héctor Eduardo Hernández-Nuñez, Isabel Gutiérrez-Montes, Juan Carlos Suárez, Hernán J. Andrade, Angie Paola Bernal Núñez, Verenice Sánchez Castillo, Gustavo Adolfo Gutiérrez, Fernando Casanoves, Cornelia Butler Flora

**Affiliations:** 1 Facultad de Ingeniería Agronómica, Programa de Doctorado en Ciencias Agrarias, Universidad del Tolima, Ibagué, Tolima, Colombia; 2 Facultad de Ingeniería, Programa de Ingeniería Agroecológica, Universidad de la Amazonia, Florencia, Caquetá, Colombia; 3 Centro de Investigaciones Amazónicas CIMAZ Macagual Cesar Augusto Estrada González, Grupo de Investigaciones Agroecosistemas y Conservación en Bosques Amazónicos- GAIA, Florencia, Colombia; 4 Doctorado en Ciencias Naturales y Desarrollo Sustentable, Facultad de Ciencias Agropecuarias, Universidad de la Amazonia, Florencia, Caquetá, Colombia; 5 CATIE – Centro Agronómico Tropical de Investigación y Enseñanza, Turrialba, Costa Rica; 6 Distinguished Professor of Sociology and Agriculture and Life Sciences. Iowa State University, Ames, Iowa, United States of America; Federal University of Agriculture and Development Studies, Iragbiji (FUADSI), Nigeria, NIGERIA

## Abstract

Rural conditions in Colombia have changed in recent years, and rural households have had to redesign their livelihood strategies (LS). Cocoa cultivation has accompanied this process of LS configuration, with a differentiated role in each household. This research analyzes the incidence of cocoa cultivation in the LS of rural households in the Colombian departments of Caquetá, Huila, Meta, and Santander. A semi-structured interview and a survey were applied in 406 rural households with cocoa production, examining the types of household LS, the specific household-level assets for cocoa production, and the perception on the representation of the crop for the households were identified. Five types of rural households were identified according to LS configuration: Cocoa Farmers, Livestock-Cocoa Farmers, Coffee-Cocoa Farmers, Diversified and Off-farm-Cocoa Farmers. It was found that cocoa cultivation has a different role and conditions in the types of households according to the LS adopted, a difference that was more accentuated at the departmental level. Households of Santander have better capital endowment for cocoa production; the opposite is the case in Caquetá. We found that cocoa production has a positive relationship (p < 0.05) with the technological level of cocoa, dedication to the crop and cocoa area. The reasons that led rural households to plant cocoa were mainly territorial conditions (not the type of rural household), economic motivation and ease of management. Finally, 90% of the households indicated that the crop has a promising future, which they associate with a livelihood, a secure source of income for old age, and the fulfillment of basic needs.

## Introduction

Colombia possesses a range favorable conditions that support agricultural development, including its diversity of thermal floors, abundant natural resources, and strategic geographical location [1]. These characteristics have led to the importance of de the rural sector in the country being recognized as a Latin American “agricultural pantry” [2], highlighting the importance of the rural sector in its economic and social development [3]. In this context, agriculture has been identified as a key sector with the potential to contribute to addressing major challenges such as food insecurity [4]. However, despite these favorable conditions, rural areas in Colombia have been historically associated with structural limitations that affect their development [5].

Despite these conditions, rural areas in Colombia have been historically associated with structural limitations such as poverty [6,7], conflicts over land use, access and tenure [8], gender inequity [9], and the effects of internal armed conflict and illicit economies [2,8,10]. In addition, rural territories face constraints related to market access and limited opportunities for decent work, particularly for rural youth [10]. More recently, emerging dynamics such as climate variability [9,11,12], changing market conditions and associative processes [13], and the aging of the rural population [14] have added complexity to these contexts.

Households in rural Colombia have adapted to these conditions by developing and modifying their livelihood strategies to ensure their subsistence and reduce vulnerability [15,16]. Cocoa cultivation (*Theobroma cacao* L.) represents a relevant component within these strategies, as it constitutes an important economic and productive activity for approximately 65,000 rural households in the country [17]. The role of cocoa in rural Colombia is therefore linked to the configuration of livelihood strategies adopted by households under different socioeconomic and environmental conditions [18].

The production of cocoa in Colombia has historically been associated with rural livelihood strategies. In the country, approximately 90% of cocoa is established under agroforestry systems (AFS) [19], which are linked to different ecological and productive characteristics. These systems have been associated with the provision of ecosystem services [20], biodiversity conservation [21,22], and livelihood-related benefits such as income generation and resilience in rural contexts [23,24]. Additionally, cocoa production is related to the generation of economic income and other socioeconomic dynamics at the household and community level [25–28].

In Colombia, different institutional and policy initiatives have been implemented to strengthen the cocoa sector [29,30]. These include national development strategies and programs aimed at promoting cocoa production and its agro-industrial development. In addition, international cooperation efforts have supported cocoa-producing regions, particularly in areas affected by armed conflict, where cocoa has been promoted within broader rural development processes [31–33].

The productive, environmental, and social conditions of cocoa production systems vary across the departments included in this study. In Caquetá and Meta, cocoa production has been influenced by processes related to the expansion of illicit crops, subsequent eradication programs, and changes associated with the peace process [34,35]. In contrast, Santander represents a traditional cocoa-producing region with a long-standing role in regional economies [30,36]. In Huila, cocoa production has shown variability over time, along with recognized potential for the development of fine cocoa [37]. These differences reflect diverse territorial contexts in which cocoa production is embedded.

The diversity of territorial contexts in which cocoa production is embedded is associated with differences in the configuration of rural livelihood strategies. Previous studies have examined rural livelihoods under conditions of environmental stress, including the assessment of livelihood risk and vulnerability to climatic hazards, the analysis of coping strategies and local responses to multi-hazard conditions, and the use of indicators to evaluate resilience capacity at the household level [38–40]. These studies highlight the importance of territorial conditions and resource availability in shaping livelihood responses. However, such approaches have not been sufficiently applied to the analysis of specific agricultural production systems, nor to the examination of how these systems are integrated within livelihood strategies across different territorial contexts.

The aim of this study is to analyze the relationship between cocoa cultivation and the livelihood strategies of rural households in the Colombian departments of Caquetá, Huila, Meta, and Santander. To address this objective, the following research questions are proposed: a. What types of livelihood strategies are identified among cocoa-producing rural households? b. What assets associated with cocoa production are used within these livelihood strategies? and c. What role does cocoa cultivation play within the livelihood strategies of rural households?

The research questions are addressed using the sustainable livelihoods approach and the community capital framework. The sustainable livelihoods approach, as proposed by Chambers and Conway [41], provides an analytical perspective to examine the relationship between livelihood strategies, resource use, and socioeconomic conditions in rural contexts [42]. The sustainable livelihoods approach has also been used as an analytical lens to examine how different forms of capital and local perceptions interact in shaping livelihood systems in rural contexts [43]. Within this framework, livelihood strategies are understood as the set of productive and reproductive activities developed by rural households [44,45]. This approach is applied in this study to analyze how cocoa production is integrated within the livelihood strategies of rural households across different territorial contexts. The result of this analysis will contribute to the integrated and sustainable management of the territory that integrates aspects of agriculture and rural resource management with human relations, forms of organization, institutions and capacities [46]. This study examines how cocoa production systems are integrated within livelihood strategies across different territorial contexts, providing empirical evidence on these interactions in rural systems.

## Materials and methods

### Study area

The study was conducted in the departments of Huila, Meta, Santander, and Caquetá in Colombia. The selection of departments was based on differences in their participation in national cocoa production and the availability of producer households, allowing the inclusion of contrasting contexts for analysis. Specifically, cocoa bean production in 2024 shows marked differences among these departments. Santander contributed 28,044 tons, ranking first in national production with 41%, followed by Meta with 3,104 tons (6%), Huila with 2,221 tons (3%), and Caquetá with 185 tons (0.28%) [17,47]. These departments also differ in the number of households engaged in cocoa cultivation and in the dynamics of cultivated areas. For example, Santander has 21,295 families and a yield of 471 kg ha^-1^ year^-1^; Huila has 2,685 producing families and a yield of 414 ha^-1^ year^-1^; Meta shows the second-highest yield in the country with 508 kg ha^-1^ year^-1^ and an average of 2.5 ha per production unit. Caquetá reports yield of 355 kg ha^-1^ year^-1^ in crops with an average size of 1.9 ha [47]. In each department, municipalities with significant participation in regional cocoa production were prioritized. Household identification was carried out using producer lists from local associations: APROCAHUILA (Association of Cocoa Producers of Huila), CACAOMET (Cocoa Producers Association of Meta), ACAMAFRUT (Departmental Association of Cocoa and Timber Species Growers of Caquetá), and FEDECACAO (National Federation of Cocoa Producers). From these lists, 406 rural households were selected for the application of surveys and semi-structured interviews [48,49]. The study population was selected using a stratified random sampling approach, considering municipalities and producer associations as strata. Within each stratum, households were selected from the producer lists provided by local associations, which were used as sampling frames to identify eligible households.

The present research was approved by the Ethics Committee of Universidad de la Amazonia, Colombia, on April 28, 2020. This approval guarantees that the research complies with fundamental ethical principles, including respect for the rights of participants, and confidentiality of information. In addition, it supports the scientific integrity of the study, ensuring that its procedures complies to national and international standards in academic research, contributing to the credibility and validity of the results obtained. In the description of results, the anonymity of each of the participants is preserved; they are mentioned in the document as “Producer n”. The data collection was carried out between November 2020 and December 2021.

### Data processing

The analyses followed a mixed-methods approach, involving the collection and analysis of quantitative and qualitative data [48]. Quantitative analyses were used to identify patterns and differences between household types and departments, including livelihood strategies and capital endowment. Qualitative analyses were used to interpret and explain these patterns through coding, categorization, and analysis of narratives derived from interviews. The integration of both approaches was carried out during the analysis phase. Quantitative results were used to structure the analysis, while qualitative information provided explanatory depth by supporting the interpretation of statistical patterns through categorical relationships and representative textual quotations. The analyses were performed using InfoStat [50], R Core Team [51], Atlas.ti version 9.1.7 [52], and UCINET 6 [53] software.

Frequency of categorical variables were generated using contingency tables [54]. Variables derived from quantitative information were analyzed as follows: a. continuous variables were treated with General Linear and Mixed Models, which allowed the consideration of heterogeneous variances between groups and the inclusion of household-level random effects [55], and b. discrete variables were analyzed through Generalized Linear Mixed Models with Poisson distribution [56]. In all cases, normality assumptions were checked. Mean comparisons were performed with LSD Fisher (p < 0.05).

### Livelihood strategies of rural cocoa-growing household types

Household livelihood strategies were classified using the characteristics of their productive livelihoods [57]. Ten quantitative variables were used, selected from livelihood classification studies conducted Bernal et al. [58]; Liyama et al. [59]; Paudel et al. [60]; Sun et al. [61,62]: (a) Ratio of coffee area/productive area of the household, (b) Ratio of pasture area/productive area of household, (c) Ratio of cocoa area to productive area of the household, (d) Ratio of other crop areas/productive area of the household, (e) Ratio of cocoa income/household income, (f) Ratio of coffee income/household income, (g) Ratio of earned income/household income, (h) Ratio of other farm income/household income, (i) Ratio of other livestock income to household income, (j) Ratio of off-farm income to household income. These variables are presented in S1 Table, including their units, definitions, and calculation procedures. They represent proportions of land use and income, allowing the assessment of livelihood diversification and dependence on specific productive activities, and have been previously applied in similar contexts [58]. In this context, “other livestock income” refers to income derived from minor livestock activities such as pigs, poultry, and fish production.

Based on these variables, a cluster analysis was performed using Ward’s method and Euclidean distance, which allowed grouping households according to similarity in their livelihood strategies [54]. Subsequently, an analysis of variance (ANOVA) was performed to estimate the variables that influenced the separation of the groups [55]. Textual quotations of the producers’ perception (active voice) were used to justify the category and type of household. The correspondence between household types and their location (department) was estimated using a contingency table analysis [54].

### Capital endowment in types of rural households for cocoa production

The capital endowment of rural households engaged in cocoa production was analyzed using the community capitals framework proposed by Flora, Flora [63]. This framework allows the assessment of assets that influence development processes by considering different forms of capital [64]. Although it has been mainly applied at the community level [65,66], it has also been used to analyze rural households and livelihood typologies [18]. In this study, the framework was adapted to the production level, specifically cocoa farming, to identify the specific assets associated with cocoa production in rural households grouped by livelihood strategies. This adaptation allows the analysis of capital endowment at the household level, linking asset availability with livelihood strategies and cocoa production dynamics.

Based on previous studies on cocoa production systems [67,68], variables related to cultural, natural, human, built, social-political, and financial capital were selected to characterize the asset endowment of rural households. Cultural capital included experience in cocoa cultivation, time devoted to cocoa-related activities, identity with cocoa production, and motivations for planting cocoa. Natural capital was represented by the area established in cocoa. Human capital included the number of training events attended and the level of knowledge about cocoa cultivation. Built capital referred to the technological level associated with cocoa production. Social-political capital was represented by participation in cocoa associations. Financial capital included annual cocoa production and income derived from cocoa.

An analysis of variance was performed to compare the endowment of cocoa assets between household types across departments (Balzarini et al., 2008). Similarly, the relationships between cocoa asset endowment variables were analyzed by generating Spearman correlations between variables in a unit-wise manner. Then, a principal component analysis (PCA) was performed using the cocoa asset endowment variables to explore the relationships among variables and identify patterns of association. The analysis was performed in R using the FactoMineR package [69], and the results were presented in the form of a biplot to facilitate interpretation.

### Cocoa cultivation: Motivations, community relations, cohesion drivers, and life projects

Four analytical dimensions were identified: households’ reasons for cultivating cocoa, cocoa cultivation and community cohesion, key drivers of community cohesion around cocoa cultivation, and cocoa cultivation and life project. For this purpose, responses were coded and categorized following an inductive approach [70]. To analyze households’ reasons for cultivating cocoa and the relationship between cocoa cultivation and community cohesion, co-occurrence matrices were constructed based on the simultaneous presence of categories within the same responses. This approach allowed the identification of associations between categories and the structure of relationships within the data, using a social network analysis approach [71].

For the identification of key drivers of community cohesion around cocoa cultivation, network representation was developed directly from the coded data, allowing the interpretation of conceptual relationships between categories. Finally, the dimension related to cocoa cultivation and life project was analyzed through the interpretation of coded narratives, focusing on the relationships between cocoa production and household-level aspirations and trajectories. In all cases, the results were complemented with representative textual quotations from the interviewees to support the interpretation of the findings.

## Results

### Livelihood strategies of cocoa producing households

Five types of rural cocoa-growing households were identified, showing statistically different representation (p < 0.05) in their livelihood strategy configuration. These typologies reflect different levels of diversification and dependence on cocoa cultivation. The identified groups were Cocoa Farmers (n = 121, 30%), Livestock-Cocoa Farmers (n = 102, 25%), Coffee-Cocoa Farmers (n = 43, 11%), Diversified Farmers (n = 88, 22%) and Off-farm-Cocoa Farmers (n = 53, 12.81%) (Table 1).

**Table 1:** Types of livelihood strategies of cocoa producing households in the study area.

Variable	Cocoa Farmers	Livestock-Cocoa Farmers	Coffee Cocoa Farmers	Diversified Farmers	Off-farm-Cocoa Farmers	P valor
Number of productive activities	1.77 ± 0.08d	2.94 ± 0.11b	3.19 ± 0.18b	3.74 ± 0.12ª	2.54 ± 0.14c	<0.0001
Ratio of coffee area/productive area of the household	0.01 ± 0.01b	0 ± 0b	0.4 ± 0.01a	0.01 ± 0.01b	0 ± 0.01b	<0.0001
Ratio of pasture area/productive area of household	0.02 ± 0.01c	0.78 ± 0.02a	0.13 ± 0.04b	0.09 ± 0.02b	0.02 ± 0.01c	<0.0001
Ratio of cocoa area to productive area of the household	0.93 ± 0.01a	0.19 ± 0.02d	0.43 ± 0.03c	0.6 ± 0.04b	0.94 ± 0.02a	<0.0001
Relationship to other areas/productive area of the household	0.05 ± 0.01b	0.03 ± 0.01b	0.04 ± 0.02b	0.29 ± 0.03ª	0.04 ± 0.01b	<0.0001
Ratio of cocoa income/household income	0.91 ± 0.01a	0.33 ± 0.03b	0.3 ± 0.03b	0.32 ± 0.03b	0.31 ± 0.03b	<0.0001
Ratio of coffee income/household income	0 ± 0b	0.01 ± 0b	0.51 ± 0.04a	0.01 ± 0b	0 ± 0b	<0.0001
Ratio of earned income/household income	0.01 ± 0b	0.39 ± 0.03a	0.05 ± 0.02b	0.04 ± 0.01b	0 ± 0b	<0.0001
Ratio of other farm income/household income	0.04 ± 0.01bc	0.02 ± 0.01c	0.09 ± 0.02b	0.29 ± 0.03ª	0.01 ± 0c	<0.0001
Ratio of other livestock income to household income	0.01 ± 0b	0.04 ± 0.01b	0.01 ± 0.01b	0.2 ± 0.03ª	0.02 ± 0.01b	<0.0001
Ratio of off-farm income to household income	0.02 ± 0d	0.21 ± 0.03b	0.05 ± 0.02d	0.14 ± 0.03c	0.66 ± 0.03a	<0.0001

Values represent the mean ± standard error. Different letters between columns show significant differences between types of rural households (p < 0.05). Green color range highlights the ratio of the areas destined for the development of livelihoods over the total area of the farm. Brown color range highlights the ratio of income derived from livelihoods in relation to total household income.

**Cocoa Farmers.** Characterized by a high dependence on cocoa cultivation, which represents 3.59 ha and 91% of the productive area (Table 1). These households show low diversification, with limited participation of other agricultural activities. The largest proportion of households in this group is located in Santander (43%) and Huila (41%), followed by Meta (12%) and Caquetá (3%).

**Livestock–Cocoa Farmers.** Characterized by a diversified income structure dominated by livestock (39%), cocoa (33%), and off-farm activities (21%). The household productive area averages 24 ha, of which 78% is allocated to pastures and 19% to cocoa cultivation (Table 1). These households are mainly located in Caquetá and Meta (32–35%), with a lower representation in Santander (10%).

**Coffee-Cocoa Farmers.** Characterized by a balanced distribution between coffee and cocoa cultivation, with average areas of 2.3 ha and 2.1 ha, representing 40% and 43% of the productive area, respectively. Income from coffee and cocoa accounts for 51% and 30% of total household income (Table 1). Most households in this group are located in Huila (81%).

**Diversified Farmers.** Characterized by a highly diversified livelihood strategy, with 49% of household income derived from activities other than cocoa, coffee, or livestock, while cocoa contributes 31% of total income (Table 1). Agricultural and livestock activities account for 29% and 20% of income, respectively. These households are mainly located in Huila (36%) and Caquetá (32%), followed by Meta (22%) and Santander (10%).

**Off-farm-Cocoa Farmers.** Characterized by a high dependence on off-farm income (66%), while cocoa contributes 31% of total household income. Despite this, cocoa occupies 2.65 ha and represents 94% of the productive area (Table 1), indicating a mismatch between land use and income generation. In 20% of these households, cocoa plantations were still in the establishment stage and had not yet entered production. These households are mainly located in Huila (38%) and Meta (35%), followed by Caquetá (15%) and Santander (12%).

### Livelihood capitals endowment associated with cocoa production

Cocoa production conditions vary across livelihood strategies and territorial contexts, with marked differences at the department level. Households in Santander present the highest levels of livelihood capital associated with cocoa production, while those in Caquetá show the lowest levels. Huila and Meta exhibit intermediate conditions, reflecting heterogeneous asset availability across the study area.

Experience in cocoa cultivation differed significantly (p < 0.05) between departments and household types. At the department level, the highest levels of experience were observed in Santander (21 years) and Huila (18 years), while households in Caquetá and Meta showed lower values, averaging six years. At the typology level, Cocoa Farmers, Diversified Farmers, and Livestock–Cocoa Farmers presented the greatest experience (13–14 years), exceeding Off-farm–Cocoa Farmers and Coffee–Cocoa Farmers (Table 2). The interaction between typology and department showed that Cocoa Farmers and Off-farm–Cocoa Farmers in Caquetá had the lowest experience (4 years), while other typologies across departments ranged between 2 and 6 years of experience in cocoa cultivation (Table 2). This reflects the presence of recently established cocoa systems in specific territorial contexts.

**Table 2:** Livelihood capitals associated with cocoa production across households types in the study area.

Capital Variable		Financial capital		Human capital		Cultural capital		Natural capital
		Income from cocoa	Cocoa dry bean production	Cocoa Trainings	Cocoa Knowledge	Dedication to cocoa farming activities	Cocoa Experience	Cocoa planted area
Typology		*(USD year*^*-1*^)	*(kg household*^*-1*^ *year*^*-1*^)	*(number)*		*(hours/week)*	*(years)*	*(ha)*
Cocoa Farmers	Caquetá	844.26 ± 235.14c	427.08 ± 111.98c	4.5 ± 0.5abc	3.75 ± 0.48ab	21 ± 7.55abc	4.25 ± 1.11de	2 ± 0.2b
	Huila	2144.02 ± 338.04b	1259.84 ± 194.02b	8.02 ± 0.13a	3.95 ± 0.11a	29.56 ± 2.19a	16.76 ± 1.84b	2.98 ± 0.25b
	Meta	2630.12 ± 534.08b	1680.67 ± 373.99b	3.13 ± 0.27abc	3.4 ± 0.17abc	18.84 ± 3.21bc	8.33 ± 0.8c	2.96 ± 0.79b
	Santander	7011.55 ± 776.9a	3718.37 ± 411.69a	3.92 ± 0.14abc	3.29 ± 0.13bc	32.69 ± 1.52a	22.13 ± 2.12b	4.49 ± 0.33a
Coffee-Cocoa Farmers	Caquetá	475.14 ± 124.86c	240 ± 60c	3 ± 0.75abc	4.5 ± 0.5a	9.5 ± 6.5b	5 ± 1 cd	1 ± 0.5b
	Huila	1015.02 ± 168.45b	599.97 ± 92.54b	3.89 ± 0.17abc	3.66 ± 0.16ab	22.91 ± 2.21abc	16.34 ± 2.05b	2.16 ± 0.18b
	Meta	1107.14 ± 624.23b	612.5 ± 317.79b	1 ± 0.67c	2.94 ± 0.05bc	12.89 ± 1.95b	2.25 ± 0.95e	1.75 ± 0.6b
	Santander	3302.63 ± 1828.95a	1600 ± 900a	2 ± 0.8abc	3.5 ± 0.5abc	18 ± 2b	42.5 ± 2.5a	2.5 ± 1.5b
Diversified Farmers	Caquetá	650.29 ± 116.22c	328.36 ± 57.54c	5.79 ± 0.18a	3.55 ± 0.16ab	14.79 ± 1.85b	7.06 ± 1.7 cd	2.27 ± 0.25b
	Huila	1243.54 ± 183.16b	728.13 ± 95.98b	9.03 ± 0.17a	4.13 ± 0.15a	24.94 ± 2.82ab	18.52 ± 2.72b	2.38 ± 0.25b
	Meta	1045.71 ± 287.34b	694.21 ± 188.73b	2.16 ± 0.26abc	3.02 ± 0.12bc	14.6 ± 2.23b	7.63 ± 2.39 cd	1.87 ± 0.32b
	Santander	4445.61 ± 1095.84a	2268.33 ± 595.35a	3.67 ± 0.34abc	3.11 ± 0.31bc	35.56 ± 3.44a	24.44 ± 5.63b	6.18 ± 1.34a
Off-farm-Cocoa Farmers	Caquetá	542.48 ± 276.54c	250.92 ± 134.4c	4.83 ± 0.41ab	2.5 ± 0.43c	21.67 ± 6.03abc	4 ± 1.63de	2.75 ± 0.76b
	Huila	1379.4 ± 368.51b	806.25 ± 209.46b	7.6 ± 0.21a	4.05 ± 0.2a	21.2 ± 2.96abc	18.8 ± 2.89b	2.29 ± 0.18b
	Meta	566.43 ± 179.06b	365 ± 133.58b	1.17 ± 0.3c	2.88 ± 0.12bc	14.7 ± 1.73b	6.22 ± 1.66 cd	2.31 ± 0.58b
	Santander	2713.03 ± 649.87a	1414.06 ± 312.62a	2 ± 0.4bc	3.38 ± 0.26abc	23.25 ± 3.76abc	14.13 ± 4.68bc	4.22 ± 0.87a
Livestock–Cocoa Farmers	Caquetá	883.99 ± 210.2b	438.09 ± 107.6b	5.42 ± 0.17a	3.67 ± 0.17ab	15.76 ± 2.12b	9.23 ± 1.72c	2.52 ± 0.27b
	Huila	2994.58 ± 1155.75b	1751.46 ± 650.62b	6.22 ± 0.2a	3.56 ± 0.2ab	22.17 ± 2.55abc	24.32 ± 3.68b	3.21 ± 0.57b
	Meta	1074.71 ± 236.74b	610.54 ± 106.35b	1.69 ± 0.2c	3.19 ± 0.13bc	17.08 ± 1.61b	5.65 ± 0.69 cd	2.61 ± 0.38b
	Santander	6999.5 ± 2521.8a	3562 ± 1225.7a	3.9 ± 0.32abc	3.7 ± 0.21ab	19.5 ± 4.09bc	24.7 ± 5.83b	5.33 ± 1.35a
P valor		<0.0001	<0.0001	<0.0001	<0.0001	<0.0001	<0.0001	<0.0001

Values represent the mean ± standard error; different letters between rows show significant differences between rural household types at the department level (p < 0.05). Dark green color indicates high values. Light green color indicates low values.

Dedication to cocoa production activities differed (p < 0.05) between departments and household types. Households in Santander and Huila showed the highest weekly dedication (30 and 25 hours/week, respectively), while Meta and Caquetá presented lower values (16 hours/week). At the typology level, Cocoa Farmers exhibited the highest dedication (29 hours/week), differing from the other typologies (Table 2). At the department level, Cocoa Farmers in Meta and Caquetá showed lower dedication (19 and 22 hours/week) compared to those in Santander and Huila, where values reached 33 and 30 hours/week, respectively (Table 2).

The area established in cocoa showed differed (p < 0.05) between household types. Cocoa Farmers had the largest area (3.6 ha), followed by Livestock–Cocoa Farmers, Off-farm–Cocoa Farmers, and Diversified Farmers, with an average of 2.8 ha, while Coffee–Cocoa Farmers showed the smallest area (2.1 ha) (Table 2). At the department level, typologies in Santander presented the largest areas, including Cocoa Farmers (4.5 ha), Diversified Farmers (6.2 ha), Off-farm–Cocoa Farmers (4.2 ha), and Livestock–Cocoa Farmers (5.3 ha), differing from the other departments where values ranged between 1.4 and 3.2 ha (Table 2).

The number of training events related to cocoa production differed significantly (p < 0.05) between departments. Households in Huila and Caquetá received the highest number of trainings (7 and 5 events, respectively), while Meta showed the lowest values (2 events). At the typology level, Cocoa Farmers and Diversified Farmers received more training (5 events) than the other household types (Table 2). The interaction between typology and department showed that Cocoa Farmers in Huila, Diversified Farmers in Caquetá and Huila, and Livestock–Cocoa Farmers in Caquetá received the highest number of trainings (5–8 events), compared to other typologies within each department (Table 2).

The level of knowledge about cocoa cultivation differed significantly (p < 0.05) between departments. Households in Huila showed the highest levels, followed by Caquetá and Santander, which did not differ from each other, while Meta presented the lowest values. At the typology level, Cocoa Farmers, Diversified Farmers, and Off-farm–Cocoa Farmers in Huila, as well as Coffee–Cocoa Farmers in Caquetá, showed the highest levels of knowledge, with values above 4 (Table 2).

Participation in cocoa associations differed between departments. Households in Caquetá and Huila had the highest levels of participation (71% and 69%, respectively), whereas Meta and Santander presented lower values (33% and 19%). At the typology level, Off-farm–Cocoa Farmers in Caquetá showed lower participation (33%) compared to the general pattern (Table 3). In Caquetá, participation is associated with strengthened social ties and collective organization: “*A core of family associativity has been achieved, there is brotherhood, social fabric and everyone believes that you can work legally”* (Producer 182, Off-Farm Income-Cocoa Farmers Caquetá). In contrast, in Santander, associative processes are limited and often reduced to formal affiliation to the National Federation of Cocoa Producers (FEDECACAO), a leading trade organization with national coverage that accredits affiliation through the cocoa card. However, this collective action shows low participation and is conditioned by leadership dynamics: “*Landazuri is very conducive to the crop, so everyone is very individual, there is no leadership, all associative processes have failed*” (Producer 297, Off-Farm Income-Cocoa Farmers Santander); “We have tried to create associations to work in community and share, but it has been very difficult” (Producer 726, Off-Farm Income-Cocoa Farmers Santander).

**Table 3:** Categorical variables associated with livelihoods strategies across household types in the study area.

Capital Variable		Social-political Capital	Built Capital			Cultural Capital					
Typology		Participation in cocoa associations	Technological level			Motivation to plant cocoa					Identity
		Yes	Low	Medium	High	1	2	3	4	5	Yes
Cocoa Farmers	Caquetá	75	50	25	25	0	25	50	25	0	100
	Huila	66	28	24	48	2	28	58	12	0	98
	Meta	26.67	20	0	80	6.67	0	73.33	20	0	86.67
	Santander	15.38	11.54	5.77	82.69	0	61.54	32.69	3.85	1.92	88.46
Coffee-Cocoa Farmers	Caquetá	50	50	0	50	0	50	50	0	0	100
	Huila	54.29	22.86	25.71	51.43	0	14.29	65.71	17.14	2.86	85.71
	Meta	25	25	25	50	0	0	50	50	0	100
	Santander	100	0	0	100	0	50	0	50	0	0
Diversified Farmers	Caquetá	67.86	50	21.43	28.57	0	32.14	42.86	21.43	3.57	85.71
	Huila	68.75	25	31.25	43.75	3.13	15.63	50	28.13	3.13	100
	Meta	15.79	31.58	15.79	52.63	0	0	73.68	15.79	10.53	100
	Santander	11.11	11.11	0	88.89	0	88.89	11.11	0	0	66.67
Off-farm-Cocoa Farmers	Caquetá	33.33	83.33	0	16.67	0	16.67	33.33	50	0	100
	Huila	85	10	30	60	0	45	45	10	0	95
	Meta	38.89	55.56	16.67	27.78	0	16.67	44.44	38.89	0	100
	Santander	25	25	37.5	37.5	0	62.5	37.5	0	0	87.5
Livestock–Cocoa Farmers	Caquetá	81.82	39.39	24.24	36.36	6.06	9.09	48.48	27.27	9.09	90.91
	Huila	79.17	8.33	29.17	62.5	0	37.5	45.83	12.5	4.17	83.33
	Meta	41.67	27.78	27.78	44.44	0	0	63.89	27.78	8.33	100
	Santander	20	0	0	100	0	70	10	10	10	70

The percentage of each category per variable is presented. The p-value was obtained using contingency tables (Chi-square statistic) for the hypothesis of equality of proportions.

The technological level for cocoa production (pruning shears, fermenters, dryers, and dry bean storage facilities) differed significantly (p < 0.05) between departments and household types. Santander had the highest proportion of households with a high technological level (81%), followed by Huila and Meta, where approximately 50% of households reached this level. In contrast, in Caquetá, 48% of households had a null technological level, of which 83% corresponded to Off-farm–Cocoa Farmers (Table 3). This same typology in Meta also showed a high proportion (56%) of households with low or null technological levels. In Santander, only Off-farm–Cocoa Farmers presented a lower proportion (37%) of households with a high technological level, while the same percentage of households was classified at the medium level and the remaining at the null level. In contrast, 100% of Livestock–Cocoa Farmers in Santander had a high technological level (Table 3).

Annual dry cocoa bean production differed significantly (p < 0.05) between typologies and departments. All household types in Santander showed the highest production levels (Table 2). In contrast, households in Meta and Huila did not differ among typologies (p > 0.05), but their production levels were significantly lower than those observed in Santander. In Caquetá, the Livestock–Cocoa Farmers typology presented the highest production, comparable to values in Meta and Huila, while the other typologies showed significantly lower production than the rest of the study cases (Table 2).

**Fig 1:**
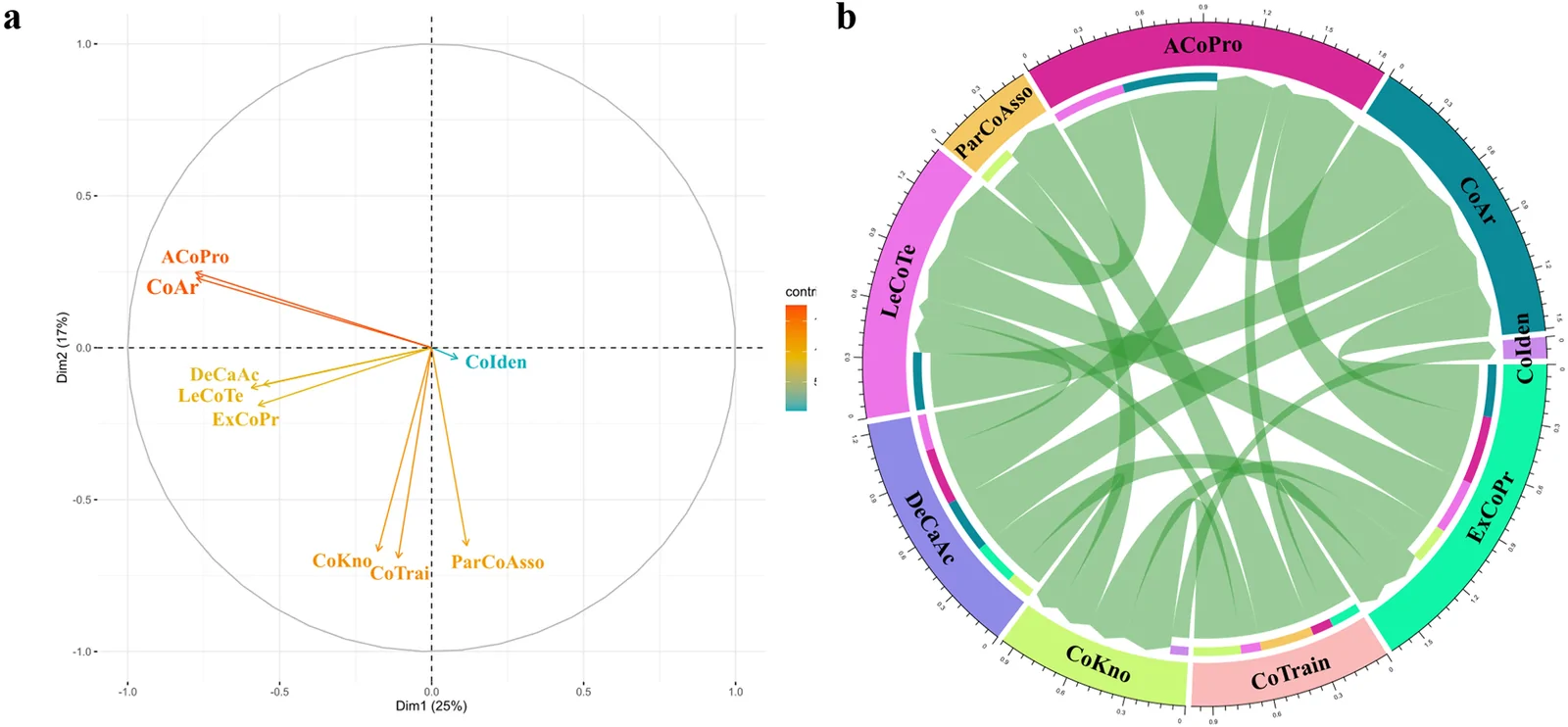
Principal component analysis of cocoa production assets across household types. a. PCA biplot of cocoa production assets. b. Correlation among cocoa production asset variables.

The principal component analysis (PCA) related the assets associated with cocoa production (Fig 1a). The first two components explained 42% of the total variance. Component 1 was associated with annual cocoa production, cocoa area, weekly dedication, experience, and technological level, while Component 2 was associated with knowledge, training, and participation in associations (Fig 1a). The variables grouped within each component showed consistent relationships. The number of trainings was positively related (p < 0.05) to participation in cocoa associations, and both variables were associated with higher levels of knowledge. Similarly, weekly dedication to cocoa cultivation was positively related to cocoa production, cocoa area, technological level, and years of experience. Finally, cocoa production was positively related (p < 0.05) to both cocoa area and technological level (Fig 1b).

## Representation of cocoa cultivation for rural households and communities

### Households’ reasons for planting cocoa crops

**Fig 2:**
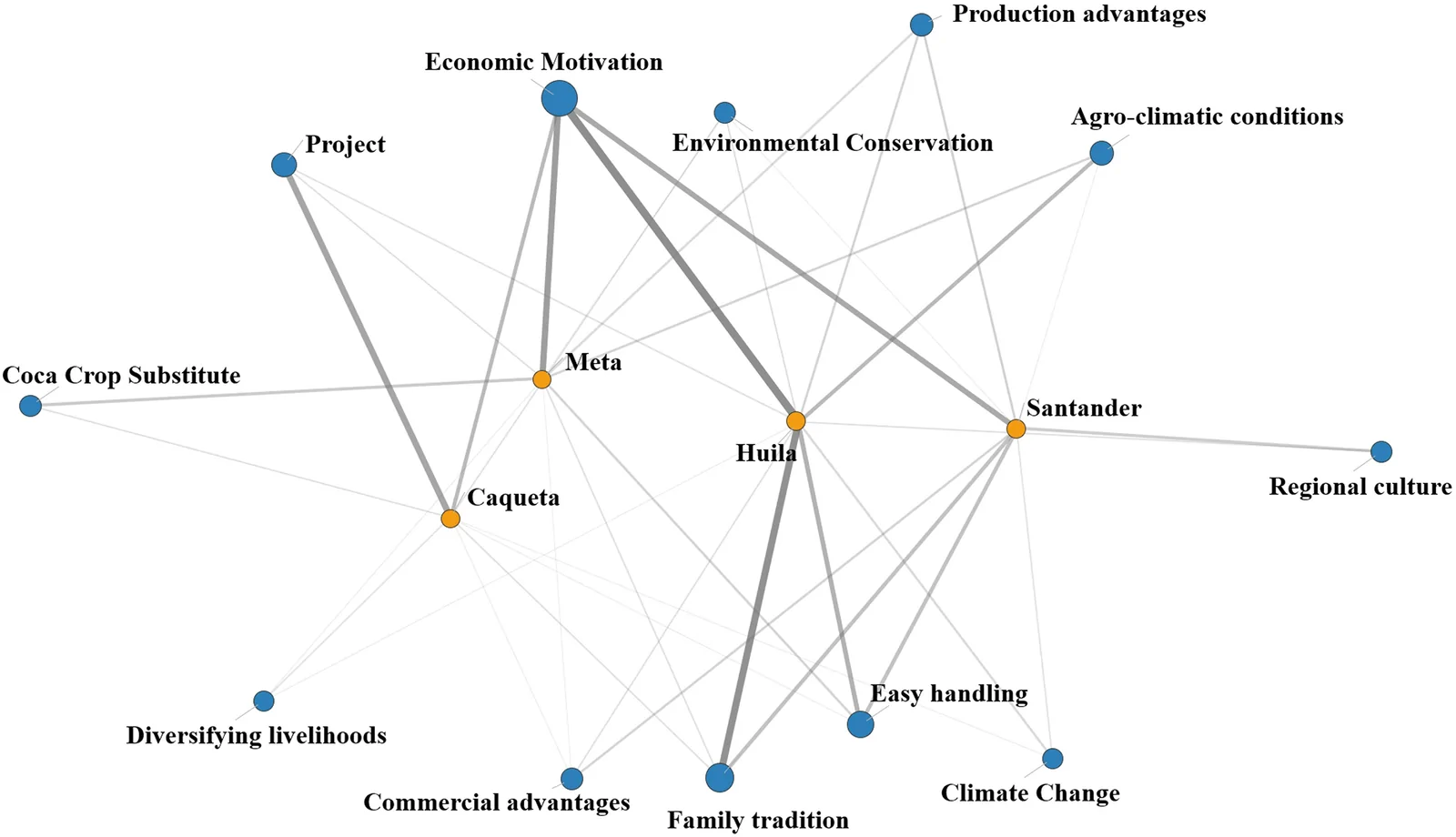
Reasons for planting cocoa among rural households in the study area. The red circles represent the departments. The blue circles represent the reasons for planting cocoa in rural households. The size of the blue circles represents the number of households reporting each reason (larger size equals more households). The thickness of the line joining the circles represents the number of households at the department level that reported each reason (larger size equals more households).

Cocoa planting by rural households was mainly influenced by territorial conditions (department) rather than household typology (Fig 2). Economic motivation and ease of management were the most frequent factors and were present across all departments. Economic motivation was reported in 36%, 27%, 25%, and 24% of households in Meta, Caquetá, Santander, and Huila, respectively, while ease of management was reported in 11%, 3%, 17%, and 15% of households in the same departments. Economic motivation was associated with constancy in production, income diversification, profitability, and favorable market conditions: “It looked like it was a plant that can be worked, and that motivated me, it is profitable and you can sell it when needed” (Producer 243, Diversified Farmers, Caquetá); “Because I needed to look for an economic solution due to the fact that employment was scarce” (Producer 411, Livestock-Cocoa Farmers, Meta); “Because it is a better profitability which leads to the satisfaction of goods and services” (Producer 351, Livestock-Cocoa–Off-farm Income Farmers, Meta).

Ease of management was related to the use of family labor, low dependence on external labor, reduced time requirements, and simplicity in harvesting and crop management: “Cocoa is one of the easiest and most prosperous crops, no worries” (Producer 130, Cocoa Farmers, Huila); “It is a crop that does not require much slavery, a manageable and sustainable crop, I like cocoa” (Producer 247, Cocoa Farmers, Santander). In several households, economic motivation and ease of management were reported simultaneously: “It is a crop that gives you time to work, it is a good independence, economically it is good, it is frequent and every 15 days you have your input for the market” (Producer 48, Diversified Farmers, Huila) (Fig 2).

The least frequent reasons for planting cocoa across all departments were family tradition, environmental conservation, and commercial advantages. In Huila and Santander, family tradition was more prominent, being reported in 23% and 17% of households, respectively: “Because that was what my husband liked, because of family tradition” (Producer 3, Cocoa Farmers, Huila); “I have been working in the cocoa system since I was 12 years old, and it is a good crop” (Producer 16, Cocoa Farmers, Huila). This motivation is linked to cultural identity in each department: “Cocoa is the flagship fruit of its region, it is easy to work, profitable, the crop is managed in the family” (Producer 300, Cocoa Farmers, Santander).

In Caquetá, although family tradition was reported in only 3% of households, cocoa was recognized as a crop with cultural and territorial roots: “Cocoa is natural gold, and it is already of descent, from our indigenous people, besides being practical for a woman to handle it” (Producer 311, Off-farm–Cocoa Farmers, Santander). In this department, the motivation “project” was more relevant, being reported in 38% of households, mainly associated with institutional support from organizations such as SINCHI, ACT, and ICA: “Here they came and motivated us, first came a project and left us that planted, but they did not return and then they fumigated and we were demotivated, but SINCHI came and helped us” (Producer 191, Diversified Farmers, Caquetá).

**Fig 3:**
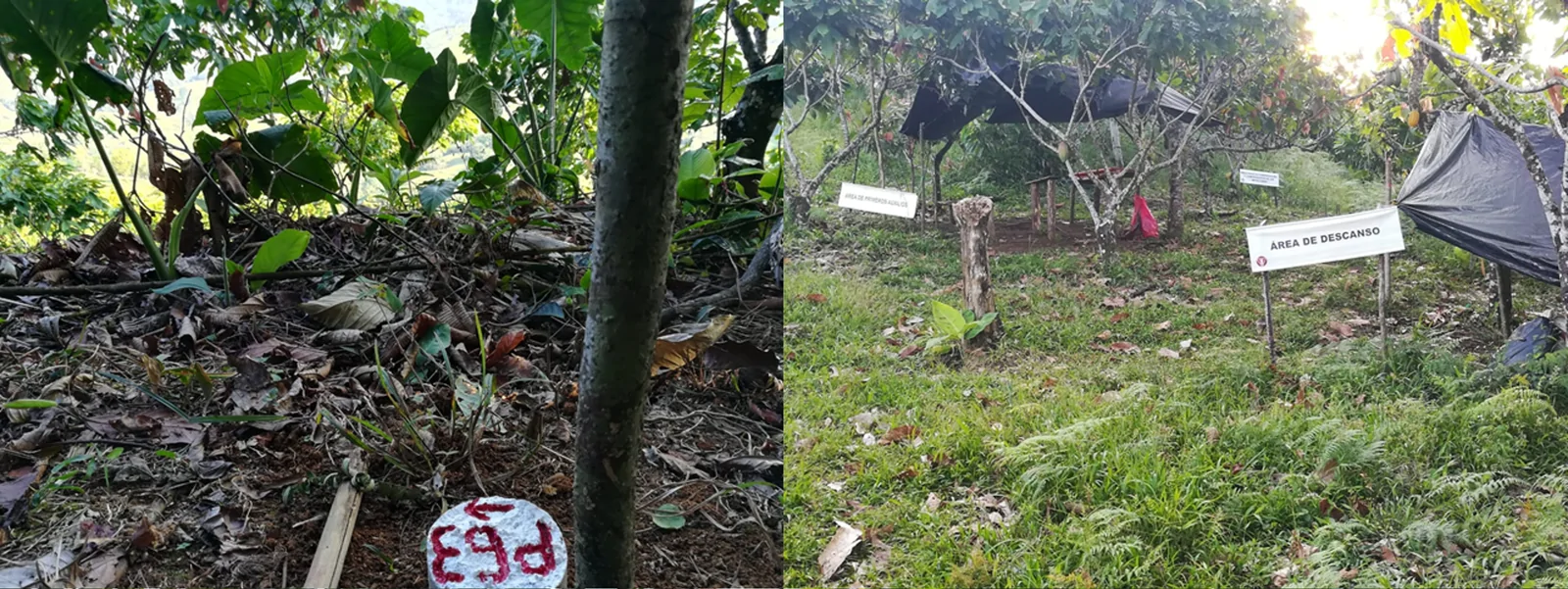
Cocoa cultivation in a former humanitarian demining zone (Caquetá, Colombia).

Cocoa cultivation was identified as a substitute for illicit coca crops in the departments of Caquetá and Meta, where this motivation was reported in 7% and 16% of households, respectively. This substitution process occurred through institutional projects or by producers’ own initiative: “A SINCHI agroforestry project. He saw that coca was no longer working and wanted to change his life” (Producer 189, Livestock-Cocoa Farmers, Caquetá); “Because it is the best solution to the problem of the previous crop (coca)” (Producer 374, Livestock-Cocoa Farmers, Meta); “Because what I had there was illegal and a lot of problems with the law, so I decided to plant cocoa” (Producer 414, Diversified Farmers, Meta). In Caquetá, some cocoa production areas located in mountainous zones have been used as logistical fields for humanitarian demining; these areas, previously affected by armed conflict, are now productive due to cocoa cultivation (Fig 3).

### Cocoa cultivation and community bonding

**Fig 4:**
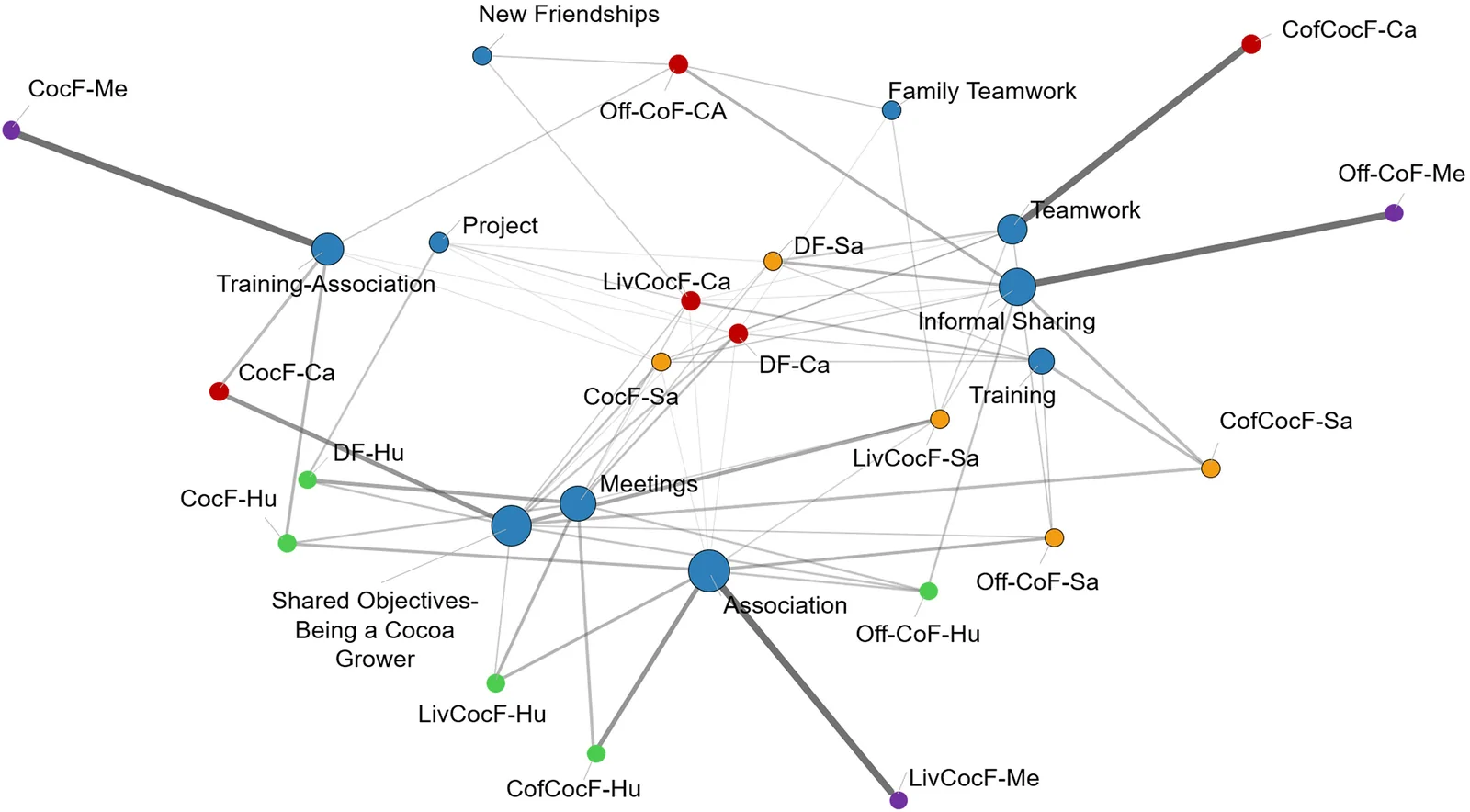
Drivers of community bonding around cocoa cultivation across household types. The blue circles represent the reasons for planting cocoa in rural households. The size of the blue circles represents the number of households reporting each reason (larger size equals more households). The thickness of the line joining the circles represents the number of households at the department level that reported each reason (larger size equals more households). Coffee-Cocoa Farmers (CofCocF), Off-farm-Cocoa Farmers (Off-CoF), Cocoa Farmers (CocF), Livestock-Cocoa Farmers (LivCocF), Diversified Farmers (DF). The letters at the end of each acronym indicate the department: Ca = Caquetá, Hu = Huila, Me = Meta, and Sa = Santander. Accordingly, different colors were used to represent each department, identified by the final letters of each typology acronym.

Cocoa cultivation was identified as a community bonding strategy associated with the activities required for its production and commercialization. The main factors for community unity included shared objectives among cocoa producers (SOCP), training processes promoted by associations and institutions, meetings organized within the territory, and daily interactions such as leisure activities (sports, drinking beer, coffee), neighbor gatherings, and informal conversations (Fig 4).

These factors showed a differentiated role in community bonding across departments. In Santander, the main drivers of community bonding were shared objectives among cocoa producers (SOCP), daily interactions, training, and teamwork, reported by 26%, 21%, 19%, and 16% of households, respectively (Fig 4). In Caquetá, a greater diversity of factors was identified, where training, SOCP, and meetings were reported by 24%, 20%, and 18% of households, respectively. To a lesser extent, factors such as associations, daily interactions, projects, and teamwork were also mentioned, along with the emergence of new friendships: “With cocoa, more people meet and talk among all cocoa farmers” (Producer 253, Off-Farm Income-Cocoa Farmers, Caquetá). In Meta, 80% of households identified associations as the main factor of community bonding, of which 20% associated this with training processes, while another 20% highlighted daily interactions as a relevant factor (Fig 4).

The factors associated with community bonding varied across household types. Shared objectives among cocoa producers (SOCP) predominated in Cocoa Farmers and Livestock–Cocoa Farmers, as reflected in the statement: “If everyone cultivates the same, they share the achievements and difficulties, they look for solutions collectively” (Producer 523, Cocoa Farmer, Santander). In contrast, association, meetings, and informal interactions were the most relevant factors in Coffee–Cocoa Farmers, Diversified Farmers, and Off-farm–Cocoa Farmers (Fig 4).

**Fig 5:**
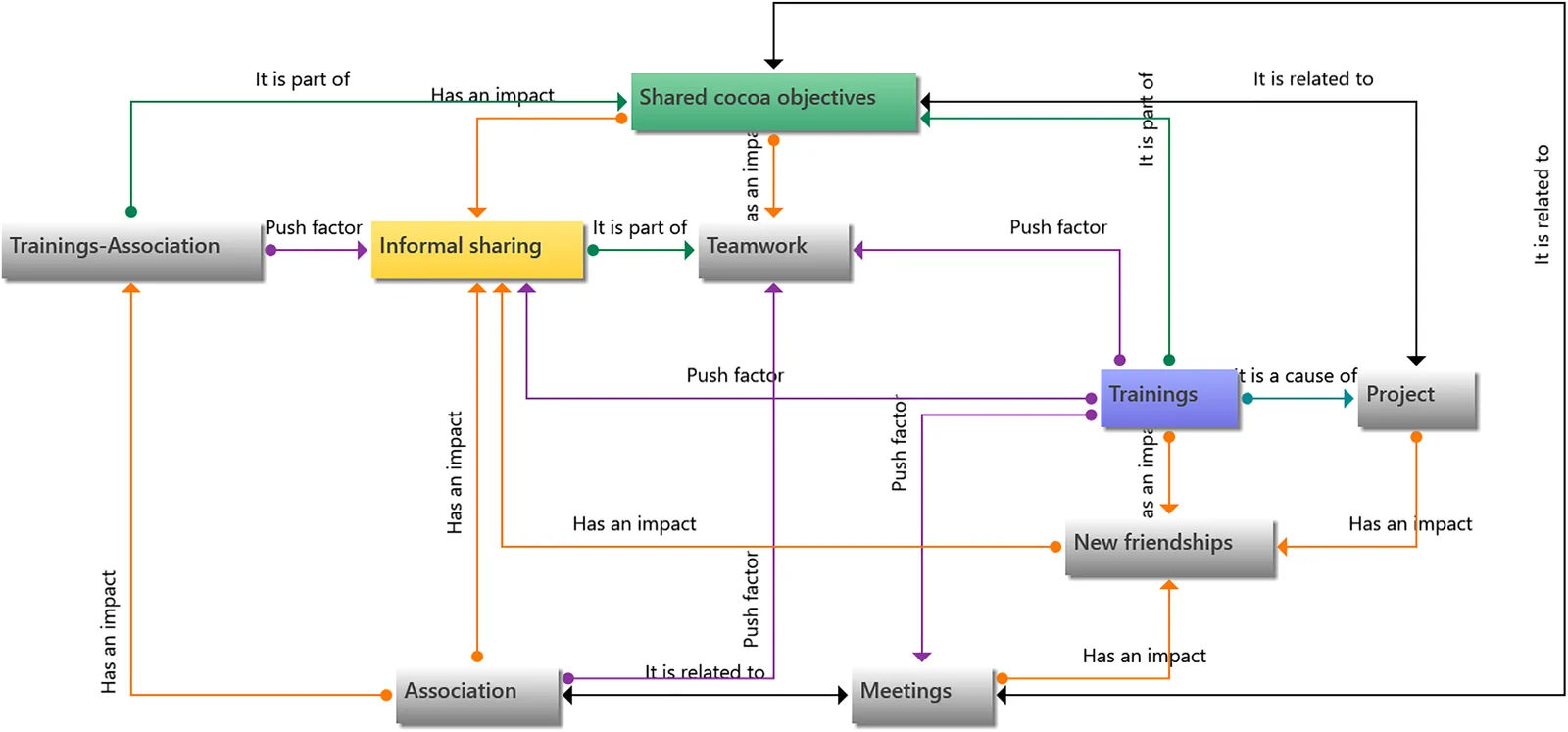
Key drivers of community cohesion around cocoa cultivation in the study area.

The interactions between factors of community cohesion showed that associations and training act as central elements that generate additional forms of social bonding (Fig 5). Households recognize associations as a key factor promoting community cohesion, as they facilitate training processes, informal interactions, teamwork, and meetings: “Yes, because of the organizations that exist, the world unites when a cup of chocolate is put on the table because it improves the living conditions of human beings” (Producer 191, Diversified Farmers, Caquetá). Associations also contribute to the articulation of leaders around common objectives: “Yes, because there are several associations in the municipalities and relationships are created even with foreigners” (Producer 309, Livestock-Cocoa Farmers, Caquetá). However, in departments such as Meta and Caquetá, the role of associations is limited by the presence of illegal groups, as collective activities around crops may generate stigmatization of producers as informants. This has led to greater marginalization of households located in mountainous areas such as San José de Fragua, El Paujil, and El Doncello (Caquetá) (Fig 5).

Training processes also act as a relevant factor of cohesion, generating spaces for interaction, knowledge exchange, and social integration: “when there are trainings they all get together and have lunch, this generates new bonds of friendship” (Producer 307, Livestock-Cocoa Farmers, Caquetá); “before I did not distinguish many people and now because of the trainings tours and meetings we are more united” (Producer 314, Livestock-Cocoa Farmers, Caquetá); “In the trainings we share knowledge, we look at other farms” (Producer 89, Cocoa Farmers, Santander); “In talks and trainings people integrate and share, we learn and improve every day” (Producer 95, Cocoa Farmers, Santander) (Fig 5).

### Cocoa cultivation and life project

**Fig 6:**
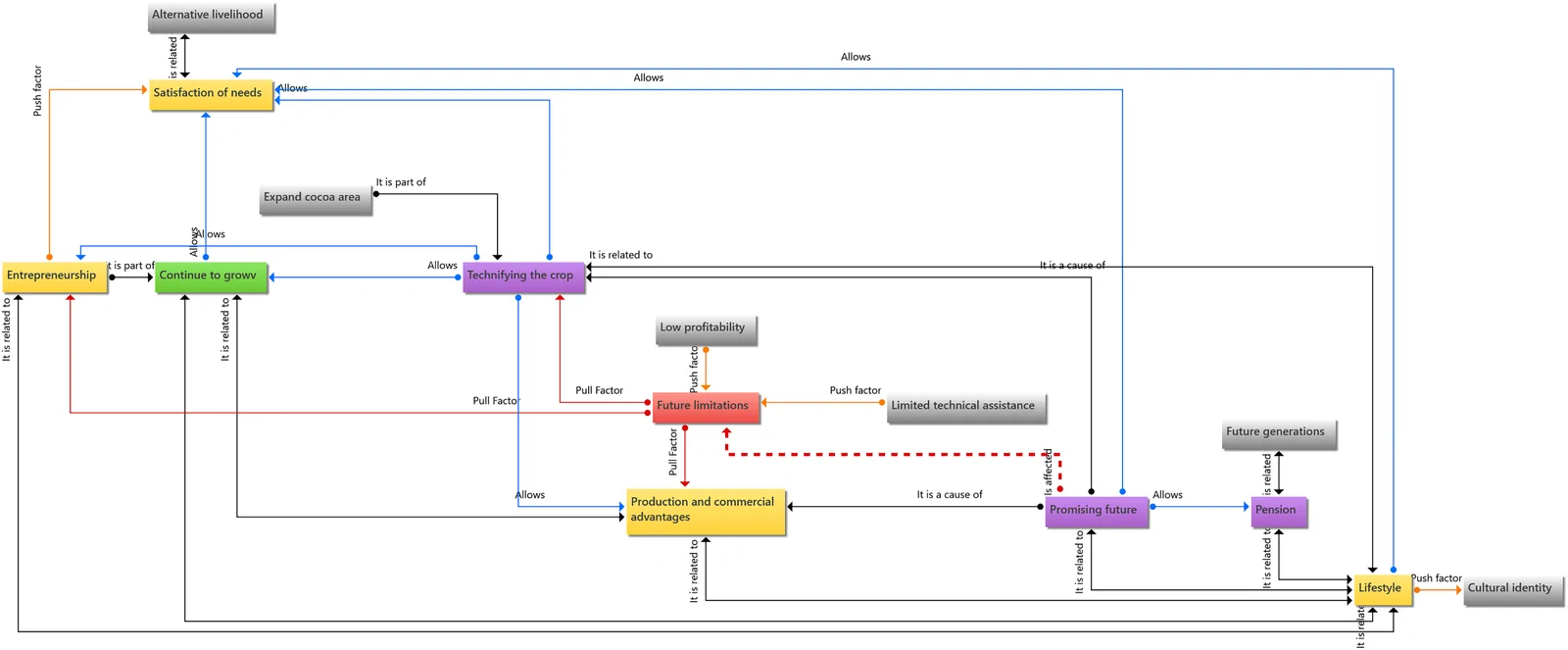
Push and pull factors linking cocoa cultivation and household life trajectories.

There are different perceptions about cocoa cultivation as a life project. Ninety percent of households indicated that cocoa farming has a promising future, which they associate with a stable livelihood, a form of pension, and the satisfaction of basic needs: “That’s what we live on, that’s how we gave our children an education” (Producer 19, Livestock-Cocoa Farmers, Huila); “If I didn’t have cocoa, life would be very hard. Cocoa is healthy” (Producer 26, Off-Farm Income-Cocoa Farmers, Huila) (Fig 6).

In this context, producers highlighted the need to improve crop conditions through technification, which would enhance production and commercialization. Some households aim to expand cocoa cultivation, while others seek to develop entrepreneurial initiatives such as processing and export: “Cocoa was my life project, the children are professionals, cocoa is a generator of peace, a generator of employment, cocoa gives a very acceptable quality of life” (Producer 80, Cocoa Farmers, Santander); “Continue with the crop and set up one’s own processing company” (Producer 240, Cocoa Farmers, Santander).

Different factors associated with cocoa as a life project are linked to cultural identity: “Being a great cocoa farmer and representing the producers” (Producer 245, Coffee-Cocoa Farmers, Santander); “It is my daily chore, I like it because it is my way of life” (Producer 110, Cocoa Farmers, Huila). Households also perceive cocoa cultivation as a long-term livelihood and a form of pension: “It is important because they say that it lasts more or less 40 years in production would be practically my pension” (Producer 98, Diversified Farmers, Caquetá).

This perception is closely related to intergenerational aspirations, as cocoa production is seen as a means to support education and improve the well-being of future generations: “Yes because when he picks the nuggets he helps the daughter and can give study to the daughter who is in 11” (Producer 252, Livestock-Cocoa Farmers, Caquetá); “It is a dream, I want to fly, to get ahead with my children and support them” (Producer 116, Diversified Farmers, Santander). However, some producers identified limitations associated with low productivity, phytosanitary problems, and constraints for crop technification, which reduce opportunities for entrepreneurship and market development (Fig 6).

## Discussion

Five typologies of rural cocoa farming households were found to have different livelihood strategies: Cocoa Farmers, Livestock-Cocoa Farmers, Coffee-Cocoa Farmers, Diversified Farmers and Off-farm-Cocoa Farmers. The diversity of livelihood strategies responds to dynamics influenced by different contextual factors, where rural households build an increasingly diverse portfolio of activities and assets in order to survive and improve their standard of living [57]. Therefore, the analysis of the strategies adopted by households must consider the real world and try to understand things from local perspectives [72]. In addition to contextual conditions, another important factor for the adoption of the rural livelihood strategy is the productive demands of the agricultural sector and the resources available to households [73]. Authors such as Anderzén, Guzmán Luna [74] have indicated that human (household composition, availability and type of labor, knowledge, skills) and financial assets are determinants in the diversification of livelihood strategies. Alonso, Delgado [75] reaffirms the importance of human capital and adds that natural and social conditions can explain the definition of a given number of livelihood strategies.

This is consistent with the livelihoods found within the strategies adopted by the households, in which cocoa cultivation, coffee cultivation and cattle ranching are more frequent activities; these respond to productive and cultural dynamics of the country and the regions; but have a different representation in each of them. In Colombia, coffee cultivation has, for many years, been one of the main products of agroindustry [76] and played an important role in economic, social and institutional development [77,78]. Livestock is another livelihood of great economic, social and cultural importance in the county [79], as Colombia is the 17th largest beef producer in the world and contributes 1.2% of the global beef supply [80]. Finally, cocoa cultivation is one of the main productive bets in Colombia due to its social and commercial potential [81], and its growing market opportunities contribute to the national economy [82], especially as a substitute for illicit coca cultivation [18,29].

The highest percentage of households corresponded to the Cocoa Farmers type, where cocoa cultivation is the most important livelihood within the household strategy. These households were found in greater proportion in the departments of Santander and Huila. This is consistent with national statistics, which by 2022 place Santander and Huila as the first and fourth largest producers of cocoa at the national level, respectively [83]. In Santander, Pabón, Herrera-Roa (81) found that 16.5% of farmers surveyed are exclusively dedicated to cocoa cultivation, while the remaining 83.5% alternate cocoa cultivation with other agricultural and livestock activities. This is consistent with the results of this research, where cocoa households receive 90% of their income from cocoa beans, but have other income-generating activities such as banana, plantain, and citrus, which are generally produced within the cocoa AFS as shade species. In other countries, the dynamics are similar: Braga, Pokorny [84] reported that in Brazil, cocoa farmers strongly focus on cocoa cultivation and rarely commercialize other crops or timber from shade trees in their AFS. In Indonesia, cocoa was the predominant crop, although several other crops generated income, including durian and rambutan (both shade trees for cocoa), cloves, coffee and rice [85].

This research found households where the livelihood strategy is supported by livestock production, cocoa, and off-farm income (Livestock-Cocoa Farmers). Most of these households were found in the departments of Meta and Caquetá. This is consistent with national statistics, where these two departments rank third and fifth in terms of livestock inventory and milk production, respectively [86,87]. However, the results of this research indicate that cattle raising is accompanied by other activities. In this regard, Braga, Pokorny [84] indicate that in households whose main livelihood is livestock raising, diversification is usually an important strategy to contribute to economic stability. Consistent with this, Hernández-Núñez, Gutiérrez-Montes (18) found that in the department of Meta, livestock farming alternates with activities such as cocoa production, which was also reported by Bernal-Núñez, Gutiérrez-Montes [58] for Huila. Globally, a similar behavior is present: in Indonesia, smallholder cocoa households diversify livelihoods with activities around livestock and off-farm activities (self-employment or wage labor) [85]. This livelihood interaction (Livestock-Cacao-culture) has been catalogued by Braga, Pokorny [84] as a livelihood strategy with a high probability of economic success and contribution to the welfare of rural families.

In the department of Huila, most households were found to have configured their livelihood strategies around coffee and cocoa. In this department, coffee cultivation is of great importance, to the point of occupying first place nationally in terms of area planted and production of coffee beans [88]. Authors have reported that, along with AFS coffee, coffee farmers carry out other agricultural activities to obtain food and income, such as raising animals or growing fruits and vegetables in home gardens [74]. This is consistent with these results, where households engage in different livelihood activities. This diversity of activities contributes to the resilience of households and socio-environmental systems, in addition to reducing vulnerability and the risk of impacts generated by dependence on growing only coffee [89]. On average, farmers reported having of 4.1 productive activities [74], where one of the determinants in diversifying the livelihood strategies of coffee farming families is natural capital [75].

Households were found to design their livelihood strategy based on the diversification of activities, where 49% of their income is derived from the cultivation of banana, banana, avocado, orange, tangerine, fish, pigs, and poultry, and 31% from the cultivation of cocoa. Diversification has been identified as an important agroecological strategy for rural development [74,89]. Irfany, McMahon (85) have indicated that diversification contributes to household wealth and welfare. In some cases, cocoa income is used as financial capital for livelihood diversification [90]. Households with livelihood diversity can improve food security, be better able to adapt to climate change, and conserve and protect natural resources [58,74]. From this perspective, household well-being is closely linked to the balance and interaction among different forms of capital, particularly human, social, and cultural capital, which play a central role in shaping livelihood outcomes [91].

Finally, there are households whose livelihoods are cocoa and work outside the household (Off-farm-Cocoa Farmers), in which 66% of income comes from external activities and 31% from cocoa cultivation. Similar results were found worldwide. In Indonesia, two key forms of external income were reported on cocoa farms: a. wage labor (including construction, farm work on other farms, and fixed contract employment) and b. self-employment income sources (e.g., running a small business such as a trading company), fresh produce, selling fish or vegetables, selling baked cakes, small truck transport, and tailoring [85]. These non-farm income sources have increased in importance in rural households [9,92]. In 20% of households in the Off-farm-Cocoa Farmers typology, cultivation had not started the productive stage. Several of these new families planted cocoa in the framework of the peace process, as a substitute for illegal coca cultivation [29]. This was confirmed by Hernández-Núñez, Gutiérrez-Montes (18) who found families in the department of Meta that they called New Cocoa Producers, several of whom replaced coca cultivation to plant cocoa.

Cocoa cultivation has a different role and assets for its production in different types of households, depending on the livelihood strategy adopted. In this regard, Román, Licea (73) indicate that each community has its own capacities, potentials and resources, which are used for its development. Therefore, the livelihoods of rural households cannot be studied separately from their environment [75], and their contribution to the household will depend on the conditions in which they operate, i.e., how the household uses its physical, economic, natural, human, social and cultural assets [89]. This is reaffirmed by different authors, who have indicated that social, economic, and ecological contextual factors affect the development of agricultural activities [74,93,94]. This is consistent with the results of the present study, where differences in assets for cocoa production have a high incidence of department; at the general level, households in the department of Santander have better capital endowment for cocoa production and Caquetá presents the highest deficiency in assets for cocoa production. However, Braga, Pokorny (84) highlight two important aspects of productive success: first, there is no single specific factor that leads to success, but rather an interaction of many factors; and second, some factors play a more important role in achieving this objective. This is consistent with what has been suggested by different authors, who indicate that there are assets that rural households have that function as a bridge to generate new assets, causing an upward spiral in the endowment of capital [18,95,96]. In this sense, recent approaches based on the community capitals framework emphasize that these assets operate as interconnected and non-linear systems, shaping the capacity of rural territories to respond to shocks and sustain development processes over time [97].

At the asset level for cocoa production, the present results indicate that experience in cocoa farming is lower in Off-farm-Cocoa Farmers and Cocoa Farmers households in the department of Caquetá (2–4 years) and higher in all types of households in Santander (14–42 years). Pabón et al. (2016) reported similar data in Colombia, finding a high percentage (61%) of producers who have been engaged in cocoa farming for more than 20 years, while 23% have been between 11 and 20 years. In Santander, Montealegre-Bustos, Rojas Molina [98] found that cocoa producers had an average of 22.6 years of experience in the crop. Although, a higher age of producers can mean a higher cultural capital [81,99], it can also represent a negative aspect from the adoption of new technologies projecting to increase the competitiveness of the sector, since younger producers are those who are open to change [73].

The area planted in cocoa crops was different at the departmental level, being greater in Santander, with plantations of between 4–6 ha; in the other departments the average area varied between 1.4 and 3.2 ha. Similar results were found by Montealegre-Bustos, Rojas Molina (98), who reported that for Santander, farmers allocate 4–5 ha to cocoa cultivation. In the same department, Pabón, Herrera-Roa (81) reported that the average cocoa plantations were 6.6 ha. Hernández-Núñez, Gutiérrez-Montes (18) state that cocoa plantations in Meta averaged 1.8 ha; however, households that depended mostly on cocoa have an average of 3.8 ha planted in cocoa.

The Cocoa Farmers in this study showed the highest dedication to crop activities, in general 29 hours per week, being different in the departments, with less dedication in Meta (19 hours/week) and Caquetá (22 hours/week) compared to the Cocoa Farmers of Santander and Huila (33–30 hours/week). The time dedicated to cultivation is one of the variables of high importance for productive development [36]. Hernández-Núñez, Gutiérrez-Montes (23) found that households with high levels of shade accompanying cocoa (income and food generator), had high cocoa bean yields when there was high dedication to the crop. However, time spent must be in synergy with other factors, such as access to appropriate technology [84], availability of inputs [100], and farmers’ experience and skills in cocoa cultivation [99].

The participation of households in cocoa associations differed between departments, being higher in Caquetá and Huila. In contrast, in Santander only 19% of households participated. Montealegre- Montealegre-Bustos, Rojas Molina (98) reported similar results for Santander, indicating that 89% of cocoa-growing households were not associated. This may be because households with greater area, production and income derived from cocoa carry out processes independently. In asset-based approaches, elements such as social networks, coordination among actors, and human capital are understood as critical resources that shape collective outcomes and the functioning of rural systems [101]. However, this is a particularity of the department of Santander. The other departments have strengths at the associative level, which has an impact on cocoa production systems. Associations offer different benefits such as access to well-established networks of donors, policy makers, researchers and private sector institutions, training and access to technologies and inputs [93]. In this regard, Braga, Pokorny [84] indicate that the level of social interactions is positively correlated with household income, security and welfare. In addition, Yiridomoh, Bonye [102] state that in the face of aspects of new rurality such as climate change, the farmer’s membership in an association indicates decisions on aspects such as adaptation to the problems generated by climate change. Therefore, it is important to promote associative processes among producers seeking to improve productive and competitive processes [103]. These dynamics are closely linked to community-level processes, where participation and human capital contribute to strengthening social cohesion, fostering involvement, and supporting broader community development processes [104].

### Policy implications for cocoa production in Colombia

The findings presented in this study have significant implications for the formulation and implementation of cocoa-related policies in Colombia. Firstly, the identification of five typologies of cocoa-producing households with differentiated subsistence strategies highlights the need to design policies tailored to the specific characteristics of each type of producer in the analyzed departments. This entails a more segmented approach that considers differences in productive assets, levels of experience, access to technology, and participation in associations according to the territorial and social context of each department. The variation in the allocation of capitals for cocoa production across departments underscores the importance of focusing efforts on areas with greater deficiencies, such as Caquetá. Policies that promote access to technology, training, and financial resources could reduce existing gaps and improve the competitiveness of producers in less advantaged regions. Additionally, strengthening producer associations in departments like Santander, where participation is low, could be key to fostering cooperation, market access, and the adoption of sustainable practices.

The role of cocoa as an alternative to illicit crops, especially in departments such as Caquetá and Meta, highlights its potential in rural development and peacebuilding programs. Cocoa policies could be integrated with initiatives for the substitution of illegal crops, promoting cocoa as a profitable and sustainable option, accompanied by technical and financial support to ensure its success. The perception of cocoa as a life project and its ability to generate stable income, improve quality of life, and strengthen the cultural identity of rural communities reinforces the need for policies that promote the modernization of cocoa cultivation and the diversification of economic activities related to cocoa. This includes encouraging entrepreneurship around cocoa processing and commercialization, as well as access to international markets. These policies should prioritize continuous training, access to inputs and technologies, and the creation of producer networks that strengthen social and community ties.

### Study limitations

The study on cocoa production in rural households presented some limitations. Firstly, geographical representativeness was a significant constraint, as although several cocoa-producing departments in Colombia were analyzed, not all cocoa-growing regions in the country were included, limiting the generalization of the findings at the national level. Moreover, data collection relied on the willingness of producers to participate, which may have introduced biases in regions where producers are less accessible or have lower participation in associations. Another significant limitation was the study’s temporal focus, based on a cross-sectional design centered on a specific period, which does not allow establishing causal relationships between cocoa cultivation and livelihood outcomes. Additionally, external factors such as recent government policies, the impact of climate change, and variations in international cocoa prices were not thoroughly addressed, restricting a complete understanding of the context in which producers operate. Furthermore, while social aspects such as participation in associations and strengthening community ties were analyzed, other social and cultural factors, such as local traditions and gender, were not included within the scope of this study, which also influenced producers’ decisions.

## Conclusions

This research manages to define five types of rural households according to different livelihood strategies and the role played by cocoa farming (Cocoa Farmers, Livestock-Cocoa Farmers, Coffee-Cocoa Farmers, Diversified Farmers and Off-farm-Cocoa Farmers). Cocoa-growing areas of different relevance in the country were studied: from those with the greatest history, culture, and cocoa production, such as Santander and Huila, to areas where cocoa is emerging and consolidating as a promising productive sector due to crop substitution programs, such as Caquetá and Meta. It is concluded that the configuration of livelihoods shows that cocoa is a crop that must coexist with other livelihoods, such as cocoa-livestock or cocoa-coffee, which is related to tradition and the dynamics of the current context.

It is shown that cocoa cultivation has a fundamental role in the development of rurality in Colombia, since it energizes the livelihood strategies of rural households and its contribution according to territorial conditions, becoming the main livelihood of households or a livelihood synergistic with other livelihoods. In addition, cocoa is a crop designed as a substitute for coca based on the history and vocation of the soil and, that today the context of peace allows free sharing “without pressures of war” helps people to unite around a crop, promoting the arrival of projects, and generating confidence for national and international investment.

Cocoa cultivation plays a different role in the five livelihood strategies found, which is mediated by its representativeness and importance to the rural household. The capacities of the households to develop cocoa farming were more influenced by the department than by the livelihood strategy. That is, the same typology has different assets for cocoa production in Caquetá, Huila, Meta and Santander, Colombia. However, at the general level, the department of Santander has better capital endowment for cocoa production and the department of Caquetá has the greatest deficiencies in capital assets.

Cocoa cultivation has different representations for households and communities, acting as an income generator that allows the development of reproductive roles and satisfies family needs. However, there are aspects that go beyond the economic, with this crop being associated with representations of living well, living happily, and living with hope. This crop allows the recognition of family participation, including children, women, men and older adults. It is recognized as a harmonious space where the daily work of farming is dignified, and as a setting for strengthening social relations and cultural identity. Finally, it is a crop that represents hope for personal, family and community development within the new dynamics of rurality.

Despite all the benefits, there are still challenges for the recognition of cocoa cultivation in the good life of farming families, as there as there remains uncertainty about the future of cocoa farming in Colombia. This specifically refers to generational replacement, agronomic limitations, and the recurring history of periods of increased interest that do not translate into continuous production or greater participation in the country’s gross domestic product. Thus, the present research can raise many more questions than those that were attempted to address. One of them being: why, despite the historical importance and all the advantages that cocoa cultivation represents, and its recognition as a promising crop, is its cultural, political and economic consolidation more limited compared to other livelihoods with less history in the country? The coexistence of livelihoods is important for generating greater welfare, but this coexistence depends on the type of livelihood. Therefore, it is important to understand which livelihoods can be combined to generate greater well-being, what combinations produce better conditions for households and communities, and what the limits of livelihood diversity are for the efficient development of agricultural activities. This considering that the best results in a livelihood are achieved when households can dedicate more time to it, have greater knowledge of it, and access better technology.

## Supporting information

S1 Table Variables measured in each family. (DOCX)
