# Supplementary material for: Cocoa cultivation as an engine of rural development: Impact on the configuration of livelihoods strategies of rural households in Colombia
Source: PLoS One. 2026 Aug 20;21(8):e0355387. doi: 10.1371/journal.pone.0355387 (PMC13492779; doi:10.1371/journal.pone.0355387)
Supplement: Supplementary file 1 [file pone.0355387.s001.docx]

**Supplementary Table 1. Variables used to characterize household livelihood strategies.**

| **Variable** | **Unit** | **Explanation** | **Determination** |
| --- | --- | --- | --- |
| Ratio of coffee area to total productive area of the household | Proportion (0–1) | Represents the relative importance of coffee cultivation in land use within the household production system | Calculated as the area cultivated with coffee divided by the total productive area of the household |
| Ratio of pasture area to total productive area of the household | Proportion (0–1) | Reflects the importance of livestock-oriented land use within the household | Calculated as the pasture area divided by the total productive area of the household |
| Ratio of cocoa area to total productive area of the household | Proportion (0–1) | Indicates the degree of land-use specialization in cocoa production | Calculated as the cocoa cultivated area divided by the total productive area of the household |
| Ratio of other crop areas to total productive area of the household | Proportion (0–1) | Captures diversification in land use through crops other than cocoa, coffee, or pasture | Calculated as the area of other crops divided by the total productive area of the household |
| Ratio of cocoa income to total household income | Proportion (0–1) | Represents the economic dependence on cocoa production | Calculated as income derived from cocoa divided by total household income |
| Ratio of coffee income to total household income | Proportion (0–1) | Reflects the contribution of coffee to household income | Calculated as income derived from coffee divided by total household income |
| Ratio of earned income to total household income | Proportion (0–1) | Represents income derived from wages or labor activities outside farm production | Calculated as earned (labor) income divided by total household income |
| Ratio of other agricultural income to total household income | Proportion (0–1) | Captures income diversification from agricultural activities other than cocoa and coffee | Calculated as income from other crops divided by total household income |
| Ratio of other livestock income to total household income | Proportion (0–1) | Represents income from minor livestock activities such as pigs, poultry, or fish production | Calculated as income from non-primary livestock activities divided by total household income |
| Ratio of off-farm income to total household income | Proportion (0–1) | Reflects the level of economic diversification beyond agricultural activities | Calculated as off-farm income divided by total household income |

All variables were calculated as proportions to standardize comparisons across households and to assess livelihood diversification and dependence on specific productive activities.
